# Live or let die: Translational insights and clinical perspectives of gasdermin B‐dependent intestinal epithelial cell fate

**DOI:** 10.1002/ctm2.787

**Published:** 2022-04-29

**Authors:** Giuseppe Privitera, Theresa T. Pizarro

**Affiliations:** ^1^ Department of Pathology Case Western Reserve University School of Medicine Cleveland Ohio USA

Our group recently reported a critical role for the lipid‐binding protein and gasdermin B (GSDMB), in intestinal wound healing,^1^ adding to a growing body of evidence indicating that GSDMB exerts important and multifaceted functions in intestinal epithelial cells (IECs). Notably, although it is well established that single‐nucleotide polymorphisms (SNPs) in *GSDMB* are associated with susceptibility to inflammatory disorders, including inflammatory bowel disease (IBD),^2^, ^3^ less is known regarding the translational relevance of these genetic variations in a clinical setting. Indeed, recent studies have been drawing increased attention to GSDMB in the field of gastroenterology because of its central role in colorectal cancer (CRC),^4^ enteric infections^5^ and IBD.^1^

The general concept of gasdermins is that they are expressed in full length (FL), inactive forms and, upon proteolytic activation, commonly mediated by a variety of proteases, their N‐terminal (NT) fragments bind to the host's plasma membrane, wherein they assemble into pores that allow the flow of ions and small proteins, and can eventually lead to a form of lytic, programmed cell death, termed ‘pyroptosis’.^6^ Functional studies of GSDMB are hindered by the absence of a mouse ortholog^6^; however, recent work has emerged to clarify its role(s) in IECs. Importantly, Zhou et al. were the first to make the compelling observation that, in CRC cells, granzyme A, derived from tumour‐resident natural killer and CD8^+^ T cells, is responsible for the proteolytic activation of GSDMB; in this model, GSDMB‐mediated pyroptosis of neoplastic clones elicits tumour‐suppressive responses within the tumour microenvironment.^4^ Hansen et al. confirm granzyme A‐mediated proteolytic cleavage of epithelial‐derived GSDMB in the context of enteric infection; however, they report the specificity of GSDMB‐NT pore assembly to the cell wall of invading Gram‐negative bacteria that induces bacterial lysis, while sparing host IECs.^5^ Intriguingly, they also observe that the Gram‐negative bacteria, *Shigella flexneri*, have evolved to evade this bactericidal function by a mechanism that involves proteasome‐dependent clearance of GSDMB.^5^

One critical feature that distinguishes GSDMB from other gasdermin family members is its ability to bind lipid membranes, both in its FL form and as NT fragments.^7^ To date, there is no evidence to suggest that GSDMB‐FL can form pores; however, our group describes a functional role for membrane‐bound GSDMB‐FL in IECs, independent of pyroptosis. Specifically, we show that in methotrexate (Mtx)‐activated IECs, GSDMB‐FL translocates to the plasma membrane, where it promotes epithelial restitution and repair by regulating cellular proliferation, migration and adhesion.^8^ These processes occur through a mechanism that is dependent on the homodimeric A chains of platelet‐derived growth factor (PDGF‐AA) that mediates phosphorylation of focal adhesion kinase (FAK), a key regulator of focal adhesions.^8^ Furthermore, we report the functional implications of two IBD‐associated *GSDMB* SNPs, *rs2305479* and *rs2305480*, showing that their presence impairs GSDMB‐dependent epithelial restitution.^1^

Collectively, this body of work constitutes an interesting paradigm of GSDMB biology in IECs, based on three cornerstones. First, GSDMB expression is inducible in IECs and is upregulated in the context of inflammation and/or carcinogenesis. Second, the activation of particular GSDMB‐dependent functions necessitates specific signals or events, often in cooperation with other gut mucosal cells and/or invading organisms. Third, GSDMB does not necessarily require cleavage to be active and can mediate either lytic or non‐lytic functions. Indeed, it is possible that two different, dichotomous pathways for GSDMB activation exist: one producing NT fragments that form pores and mediate lytic cell death (of either human or bacterial cells), and the other relying on GSDMB‐FL translocation to the plasma membrane that promotes epithelial motility. Notably, as opposed to IEC‐derived GSDMB‐FL function(s), the contribution of non‐IEC effector cells (e.g. cytotoxic lymphocytes) is paramount for GSDMB‐NT activation and subsequent IEC lysis. As such, close contact between IECs and these secondary effector cells is required to allow the interaction and direct passage of proteins from the latter to target IECs and represents a fundamental mechanism for GSDMB‐dependent lytic function.

Although seminal work on GSDMB biology has been performed for the last few years, specifically in IECs, future research is warranted to elucidate the precise mechanism(s) by which GSDMB is activated to fulfil a specific purpose in a particular setting. In this context, it is critical to investigate how the ‘direction’ of GSDMB activation is controlled (i.e. pore‐forming vs. pro‐restitution); moreover, because both GSDMB‐FL and ‐NT localize to lipid membranes to exert their functions, their cooperation with partner molecules, primarily chaperones that mediate protein translocation, represents another gap in knowledge. The downstream effectors of GSDMB‐dependent non‐lytic functions also constitute a ripe area of future studies. In this regard, and contrary to cell lysis, for which GSDMB‐NT represents the final effector molecule, the biological activity of GSDMB‐FL in epithelial repair involves a complex downstream pathway, which is partially attributed to FAK phosphorylation, but likely involves other proteins. Finally, the influence of GSDMB SNPs on susceptibility to proteolytic cleavage is likely relevant to its function, similar to what is shown for an asthma‐associated SNP^9^ and suggested for SNPs associated with susceptibility to Crohn's disease.^10^

The evidence presented so far highlights the overall protective effects of IEC‐derived GSDMB in IBD, CRC and enteric infection. However, it is remarkable to note that two opposing functions (i.e. proliferation vs. pyroptosis) are proposed to mediate both effects. One could argue that, while GSDMB promotes protection in a specific disease setting through one of its functions, the opposing function might play a pathogenic role in the very same condition. For example, as pyroptosis is a form of pro‐inflammatory cell death, the possibility of GSDMB‐mediated pyroptosis promoting intestinal inflammation in IBD certainly needs to be considered; on the other hand, it is plausible that GSDMB‐dependent regulation of cell proliferation and adhesion/migration might also play a role in tumour progression. Even more compelling is the possibility that, because uncontrolled colonic inflammation significantly increases the risk of developing CRC in IBD,^11^ uncontrolled, over‐activation of GSDMB‐FL may facilitate the transition from chronic inflammation to carcinogenesis, for example in the case of colitis‐associated CRC. Finally, recent studies show that the tumour microbiomes of different cancers contain tumour‐specific intracellular bacteria.^12^ Although little is known regarding its specific presence in IECs,^13^ it is worth considering whether GSDMB‐mediated killing of intracellular bacteria might also contribute to shaping this intracellular microbiome.

It is also important to note how the aforementioned information can be parlayed into clinically relevant tools for either prognostic or therapeutic purposes. Certainly, GSDMB protein levels in the gut mucosa can be proposed as a biomarker for monitoring the disease progression of IBD and/or CRC. Indeed, the expression of gasdermins, particularly gasdermin E, has already been found to correlate with clinically relevant outcomes in CRC patients.^14^ Given the apparent dichotomous roles of GSDMB in IECs, it is conceivable that the ratio between the FL and NT forms, rather than the overall protein levels, may represent a more meaningful clinical biometrics to assess whether GSDMB primarily functions as an inducer of epithelial restitution or rather, as a pyroptotic effector (Figure 1). In this regard, it is important to point out that six splice variants are reported for GSDMB; of these, isoform 5 consists only of the C‐terminal (CT) domain,^15^ with putative inhibitory function against NT fragments. To date, the specific expression and relevance of GSDMB isoform 5 in the gastrointestinal tract have not been explored; however, it is tempting to speculate that regulatory mechanisms may exist that can upregulate isoform 5 to counteract the pyroptotic effects of GSDMB‐NT. The relative levels of GSDMB (FL‐to‐NT ratio and CT levels) might therefore help to predict the clinical course of disease, and their changes after the initiation of treatment might represent an early indicator of long‐term response to therapy. On a related topic, another area to explore is whether monitoring GSDMB levels would be useful in personalized medicine by helping one to identify patients who are more likely to respond to specific therapeutic agents.

**FIGURE 1:**
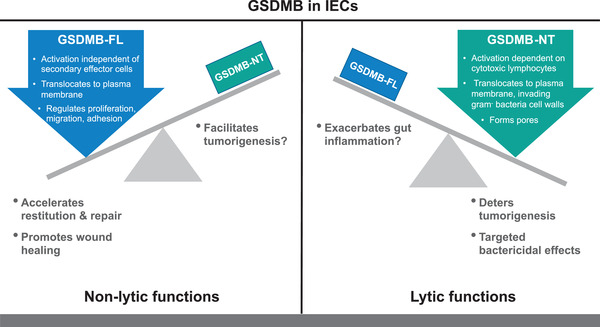
Dichotomous roles of GSDMB in intestinal epithelial cells. The overall functional effects of GSDMB in the gastrointestinal tract are that of protection, but by opposing, dichotomous roles. In IBD, upregulation and activation of GSDMB‐FL in IECs induce its translocation to the plasma membrane, regulating proliferation, migration and adhesion processes (*left panel*). These non‐lytic functions have a global effect of promoting epithelial restitution and repair that is critical for mucosal wound healing in IBD; however, if uncontrolled, these same processes may also potentially facilitate tumorigenesis. Conversely, the generation of GSDMB‐NT during either CRC or enteric infections is dependent on secondary effector cells (e.g. cytotoxic lymphocytes) for pore formation directed towards either host IECs (i.e. pyroptosis) or intracellular bacteria (i.e. bactericidal effects) (*right panel*). Although these lytic functions promote anti‐tumorigenic and targeted, Gram‐negative bacterial killing, they may also exacerbate intestinal inflammation by compromising epithelial barrier function. CRC, colorectal cancer; GSDMB, gasdermin B; GSDMB‐FL, full‐length gasdermin B; GSDMB‐NT, gasdermin B N‐terminal domain; IBD, inflammatory bowel disease; IEC, intestinal epithelial cell

Finally, in regard to the therapeutic potential of regulating epithelial GSDMB, one must consider how best to skew GSDMB towards the desired function; namely, pyroptosis of tumour cells in CRC to arrest carcinogenesis, bactericidal effects during enteric infections of intracellular pathogens or epithelial restitution in IBD to promote wound healing. In this regard, a compelling case could be made for Mtx, an immunomodulatory drug that primarily works by suppressing inflammatory functions of T‐lymphocytes.^16^ Significantly, Mtx is approved for the treatment of Crohn's disease^17^, ^18^ but failed randomized controlled trials in ulcerative colitis^19^, ^20^; these inconsistencies are difficult to reconcile, considering its proposed role in GSDMB‐mediated epithelial restitution. Nonetheless, two possible explanations can account for this outcome in ulcerative colitis: Mtx is unable to induce the adequate activation of IEC‐derived GSDMB in vivo, or its action is masked by its effects on the immune compartment. As such, it is likely that, in order to effectively exploit the therapeutic potential of GSDMB, targeted therapies that specifically regulate its expression and/or activation will be required.

In conclusion, although we are at the advent of discovery regarding the characterization and function of GSDMB, much has already been uncovered concerning its crucial role in IECs, particularly during gastrointestinal health and diseases. Future studies are greatly anticipated and are likely to reveal novel functions, as well as potential strategies to regulate the expression of this enigmatic molecule.

## FUNDING INFORMATION

This work was supported by grants from the National Institutes of Health: DK125293, AI141350, DK091222 and DK042191 (to *TTP*).

## CONFLICT OF INTEREST

The authors declare that the research was conducted in the absence of any commercial or financial relationships that could be construed as a potential conflict of interest.
